# The technique of story-telling in thyroid diseases including surgery; useful or not

**DOI:** 10.1016/j.amsu.2019.03.006

**Published:** 2019-04-13

**Authors:** Mayilvaganan Sabaretnam, Sapana Bothra, Dabeer warsi

**Affiliations:** Department of Endocrine Surgery, Sanjay Gandhi Postgraduate Institute of Medical Sciences, Raebareily Road, Lucknow, 226 014, India

**Keywords:** Story telling, Thyroidectomy

## Abstract

**Introduction:**

In the modern day busy clinical practice, the communication between patient/relative and caregiver is at a minimal level. The patients and relatives feel apprehensive when advised about surgical/interventional treatment. Storytelling is such a technique of health communication made in common man language and can operate in a virtual environment. This study aims to unveil the efficacy of storytelling technique on patients undergoing Hemithyroidectomy for benign cytology.

**Materials & methods:**

A story of a lady (cartoon version), aged 25 years, with a benign solitary thyroid nodule (STN), who underwent uneventful hemithyroidectomy was depicted in this movie including the history, clinical examination, investigations, counseling, and the operative procedure, and the running time of the animation movie is 4 min. For developing this movie, high-end graphic work station and various multimedia authoring tools like Adobe Flash, Photoshop, Captivate, Maya and Final Cut Pro, were used. The story was shown to patients with clinical STN who were provisional candidates for surgery. The patients filled in the evaluation of multimedia animation questionnaire at the time of discharge.

**Results:**

60 patients participated in the study. One form was disqualified due to incomplete filling. Mean age was 35.45 ± 12.8 years.55 (91.6%) were females. All patients were euthyroid. The mean weight of thyroid nodule was 40.80 ± 20.79 g. The final histopathology was colloid in the majority. All participants found the movie useful. In the questionnaire, the mean score for improved understanding of the disease was 73.9 ± 14.7, better organization of treatment was 78.6 ± 13.1 stimulated interest in the relatives was 70.8 ± 15.8 and saved unnecessary discussion with the consultant was 55.5 ± 7.8.

**Conclusion:**

Story telling is a useful tool in health communication. With the widespread availability of high-speed internet and affordable mobile computing devices, story telling can be a useful tool to patients and relatives in decision making and in addition, saves valuable time of the treating consultant.

## Highlights

• Addresses Patient perspective regarding Thyroid disorders.
• Need of the hour.
• Can be reproduced in any setting.
• Can be used for any disease or disorder.

## 1 Introduction

In the modern day's busy clinical practices, the communication between patient/relative and caregiver is at a minimal level. The average communication time is about 5–8 min in Great Britain and 10–20 min in The United States and Sweden [1]. Therefore, the patients and relatives feel apprehensive when a surgical/interventional treatment is advised. However, story telling such a technique may be useful in solving these issues.

### 1.1 Story telling

Story telling is the conveyance of events in words, images and/or videos (motion pictures), often by improvisation or embellishment. It is shared in every culture as a means of entertainment, education, cultural preservation, and instilling moral values. The crucial elements of story telling include plot, characters, and narrative point of view. GIVING BAD NEWS, changing from curative to palliative care, managing pain, dealing with the family of the dying patient and accepting death are some of the most difficult aspects of palliative care. They are also difficult to teach since teachers and students are experienced at imparting and receiving factual knowledge but are at a relative loss when it comes to transferring the fate of patients in our profession [2].

Supplementary video related to this article can be found at https://doi.org/10.1016/j.amsu.2019.03.006.

The following are the supplementary data related to this article:

Video 1 Video 1

One of the most powerful and time-honored teaching tools is the sharing of personal experience in the form of stories. Many important facets of palliative care such as morals and ethics, the importance of touch, and the impact of the provider-patient relationship, are not easily taught by conventional methods. Stories can provide an approach to subjects that might otherwise be uncomfortable, cause defensiveness in the learner, or be relegated to the optional curriculum because they are affective rather than cognitive [[2], [3], [4], [5]].

Stories that are effective as tools in clinical teaching differ from case presentations in that they are affective rather than cognitive. Oral stories are vivid and memorable, with each story providing context meaning, and a rich source of personal associations that facts and data alone cannot convey. To be effective as tools for teaching values and attitudes, stories must match the needs of the learner with the topic by focusing on professionalization and or the patient-provider relationship, and they must be told at the teachable moment [[2], [3], [4], [5]].

### 1.2 Aim and objective

To design and develop Story telling module for Thyroid Diseases and also evaluate the efficacy of the model using modified multimedia Animation Questionnaire.

## 2 Materials and methods

**Fig. 1:**
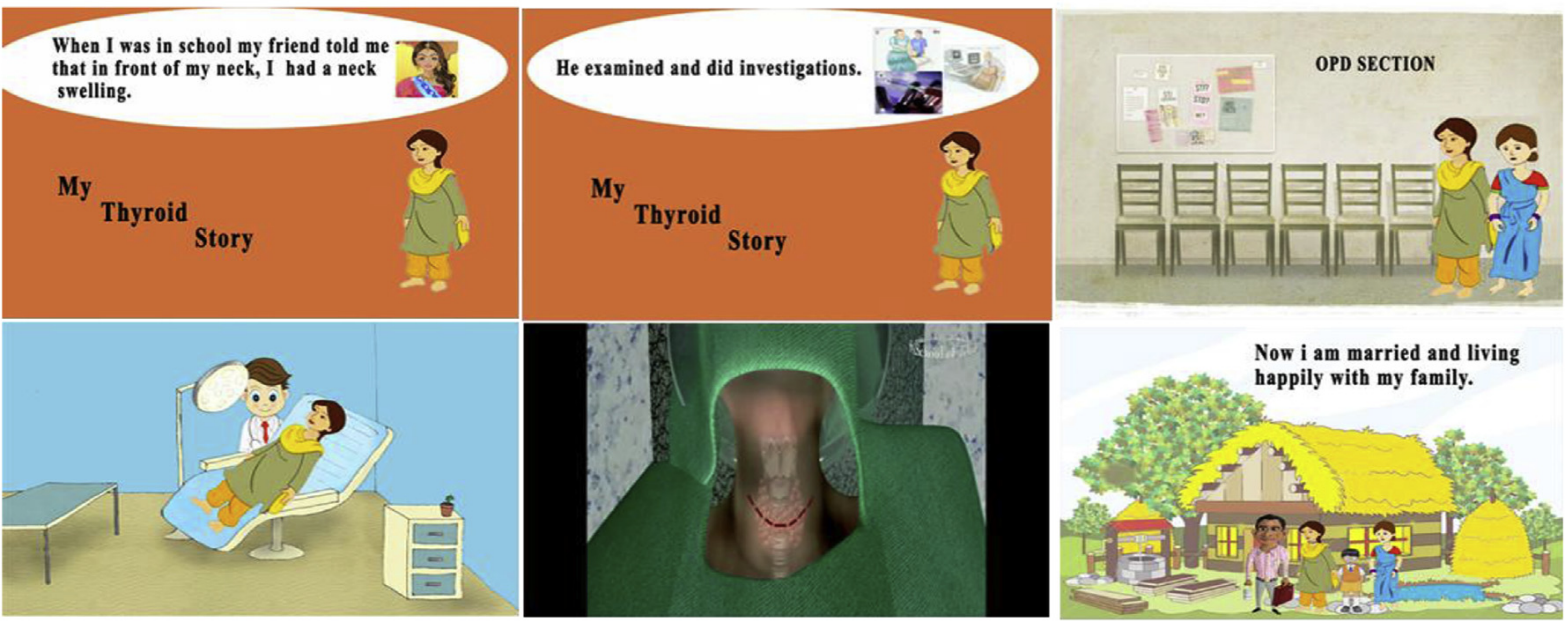
Clippings from video.

**Fig. 2:**
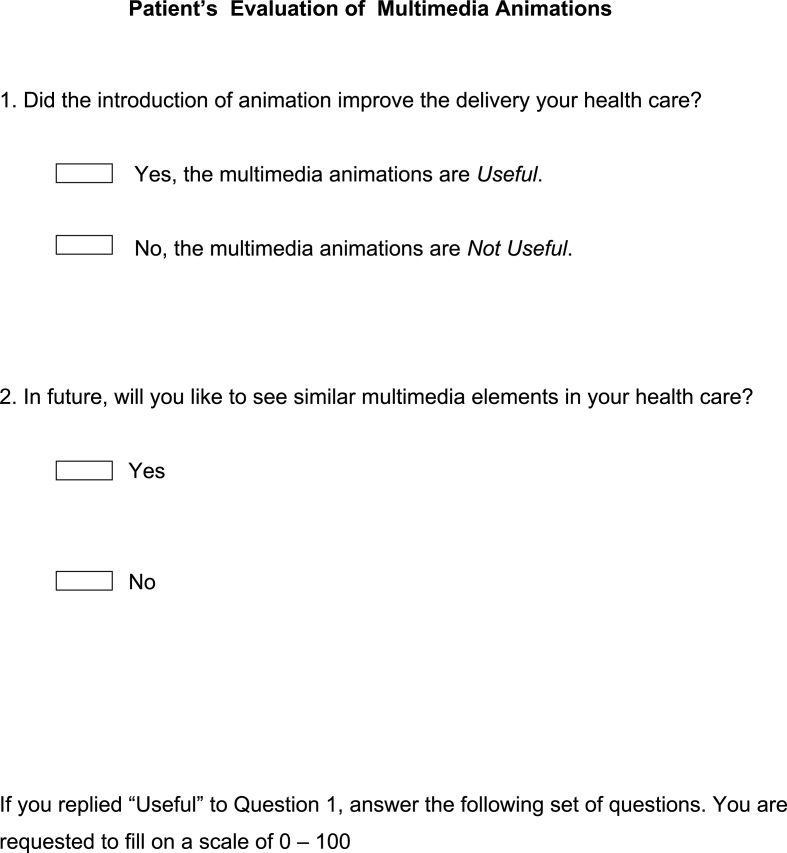
Questionnaire.

A story of a 25 years old lady, Sona, (cartoon version) with a benign thyroid nodule, who underwent uneventful hemithyroidectomy was depicted in this movie including the history, clinical examination, investigations, counseling, and the operative procedure and the outcome. Running time of the animation movie is 4 min. The young lady in this animation movie has a solitary thyroid nodule which on investigation was benign or indeterminate [6] and the patient underwent uneventful hemithyroidectomy [7], and in the follow up has normal life style. The video also has the aspect of young lady getting married and giving birth to children to allay the fear associated with these thyroid problems especially in rural women. For developing this movie, high-end graphic computer work station and various multimedia authoring tools like Adobe Flash, Photoshop, Captivate, Maya and Final Cut Pro were used. The story was shown to patients with clinically solitary thyroid nodules who were provisional candidates for surgery in the outpatient department of Endocrine Surgery in a tertiary referral centre between June 2015–June 2016. This study was approved by the ethics committee. The patients filled in the evaluation of multimedia animation questionnaire at the time of discharge. The animator was trained in medical animation for a period of 5 years. The Character and Questionnaire are provided in Fig. 1 and Fig. 2. This work has been reported in line with the STROCSS criteria [8].

## 3 Results

60 patients filled the questionnaire. 59 found the movie useful and their remaining questionnaire was analyzed. A-30-years-old male doctor did not fill the questionnaire. Mean age was 35.45 ± 12.8 years. 55 (91.66%) were females. All patients were euthyroid. The mean weight of the thyroid nodule was 40.80 ± 20.79 g. The final histopathology was colloid in the majority. In the questionnaire, the mean score for improved understanding of the disease was 73.9 ± 14.7, better organization of treatment was 78.6 ± 13.1, stimulated interest in the relatives was 70.8 ± 15.8 and saved unnecessary discussion with the consultant was 55.5 ± 7.8. When female and male patients were compared there was only significant difference in the stimulated interest in the relatives domain (64.71 ± 13.71 vs 88.00 ± 4.47 p = 0.002) (Table 1). The mean Body mass index of the patients were 24.7 (range: 17–31). Mean interview time was 3.23 ± 1.22 min (range: 2–6). 22 patients took 2 min to fill this questionnaire. Mean time taken by male and female respondent was not significantly different to each other's (3.40 ± 1.14 vs 3.21 ± 1.24, p = 0.754). Similarly mean difference in time score between the illiterate, below 10th standard, 12th, graduate post graduate and others (3.08 ± 1.26 vs. 2.33 ± 0.58 vs 3.10 ± 1.10 vs 2.82 ± 0.88 vs 4.0 ± 1.37, p = 0.027) was statistically significant. Multiple comparisons showed that mean difference was significant between the one pair (12th and graduate and above, p < 0.05) only rest other pairs, mean difference not significant (p ≥ 0.05). Distribution of the education (10th and above) in the male and females (80% vs. 72.8%, p = 0.276).

**Table 1:** Age, Weight and questionnaire response of total cohort, males and Females.

Variable	Total cohort	Male (N = 4)	Female (N = 55)	p value
Age in years	35.45 ± 12.8	34.60 ± 10.74	38.27 ± 11.46	0.538
Weight in gm	40.80 ± 20.79	41.28 ± 23.59	40.60 ± 18.60	0.995
Improved Understanding	73.92 ± 14.72	73.00 ± 19.87	74.29 ± 13.42	0.873
Better organization	78.65 ± 13.18	71.00 ± 17.46	81.43 ± 10.46	0.127
Simulated interest	70.81 ± 15.82	88.00 ± 4.47	64.71 ± 13.71	0.002*
Saved unnecessary discussion	55.52 ± 7.82	57.00 ± 9.75	55.00 ± 7.34	0.636

Independent sample *t*-test used.

*p-value < 0.05 is considered to be statistically significant.

## 4 Discussion

Very few story telling studies have been published in previous literature, but there are none pertaining to endocrine surgery. In our study, we found that the patients and relatives had a good understanding of the impact of hemithyroidectomy. However, in a developing country like ours, the patient and relatives always rely on the treating physician and his views. This topic of story telling present a unique challenge in that the development and pilot testing of the stories and how they are packaged is a critical step. There are various characteristics of the intervention that may influence its effectiveness, such as the readability and level of language, length and format, writing style, and capacity for emotional engagement. Another challenge is that there are numerous aspects of the interventions that can be varied, such as the medium of delivery (e.g., booklets, video, computer) and presentation (e.g., illustrations, images, colours, shape, and size). We feel researchers need to consider the optimal design of the intervention, and these attributes may shift in response to the audience, clinical condition, and end goal. We developed our intervention through an interactive process, which involved pilot testing among healthcare professionals for content validity and focus groups of patients for appeal and readability. We found this character whom we named Lady Sony depicted a rural background traditional Indian girl and so the patients felt that one of their relatives or friends is addressing the patient. The investigations and the surgical procedure also was discussed in detail to make the prospective surgical patient more familiar. We tried addressing few of the myths associated with thyroid surgery and we showed that Lady Sony had a Happy Married life with a child. In rural India women associate thyroid disorders with weight gain and some women associate all infertility issues to thyroid disorders. This results in delay in seeking treatment and majority of our patients present with goiter more than 3 cms.

In select countries like South Korea due to regular screening the thyroid disorders have become epidemic [9]. So, the busy clinician has to have such animation videos in his armamentarium to help patients under their disease without undue fear and also undergo successful completion of their treatment with minimum discomfort to both the Physician and Patient.

The myths associated with treatment of thyroid disorders are varied and also myth regarding thyroid disorders. Patients resort to many such treatments including Ayurveda, Siddha and other native treatments including tattooing before approaching an endocrinologist or an endocrine surgeon [10]. Even thyroid disorders are dealt by different doctors in India like endocrinologists, physicians, ENT surgeons, Head and neck surgeons, General surgeons and Endocrine surgeons which can result sometimes in difficult decision making for the patient and family when they seek two different opinions. So if teaching materials are available for patients and relatives which are standardized by respective societies then the decision making and become much simpler and the effort for the busy physician is one time affair.

Story telling is a useful tool in health communication. With the widespread availability of high-speed internet and affordable mobile computing devices [11,12], such kind of information can be of use to the patients and relatives in decision making and can also save valuable time of the treating consultant. Future studies with larger numbers are needed and societies should develop these standardized teaching modules for aiding decision making.

## Provenance and peer review

Not commissioned, externally peer reviewed.

## Ethical approval

Department ethics committee no PGI/549/2014.

Dated 16.09.2014.

## Conflicts of interest

No.

## Sources of funding

No.

## Author contribution

Authors’ contribution: S.M. and SB contributed to the conception and design of the study. S.M., SB., and DW did the acquisition of data. S.M., DW and SB. did the analysis and interpretation of data. S.M. drafted the article. All authors revised the article critically for important intellectual content and also the final approval of the version to be submitted.

## Research registry number

researchregistry4501.

## Guarantor

Dr. Mayilvaganan Sabaretnam.
